# Identification of miRNA-mRNA Regulatory Networks Associated with Diabetic Retinopathy using Bioinformatics Analysis

**DOI:** 10.2174/1871530323666230419081351

**Published:** 2023-10-11

**Authors:** Weihai Xu, Ya Liang, Ying Zhuang, Zhilan Yuan

**Affiliations:** 1 Department of Ophthalmology, The Binhai County People’s Hospital, Yancheng, 224500, China, 210029;; 2 Department of Ophthalmology, The First Affiliated Hospital of Nanjing Medical University, Nanjing, 224500, China;; 3 Department of Stomatology, the Binhai County People’s Hospital, Yancheng, China, 224500

**Keywords:** Diabetic retinopathy, microRNAs, ATP synthase, let-7a-5p, pathogenesis, diabetes mellitus

## Abstract

***Introduction*:** Diabetic retinopathy (DR) is a major complication of diabetes and a leading cause of visual loss. This study aimed to explore biomarkers for DR that may provide additional reference to DR pathogenesis and development.

***Methods*:** The differentially expressed genes (DEGs) between the DR and control samples in the GSE53257 dataset were identified. Logistics analyses were performed to identify DR-associated miRNAs and genes, and correlation analysis was performed to determine the correlation between them in GSE160306.

***Results*:** A total of 114 DEGs in DR were identified in GSE53257. Three genes, including ATP5A1 (down), DAUFV2 (down), and OXA1L (down), were differentially expressed between DR and control samples in GSE160306. Univariate logistics analysis identified that ATP5A1 (OR=0.007, *p* = 1.40E-02), NDUFV2 (OR = 0.003, *p* = 6.40E-03), and OXA1L (OR = 0.093, *p* = 3.08E-02) were DR-associated genes. ATP5A1 and OXA1L were regulated by multiple miRNAs, of which hsa-let-7b-5p (OR = 26.071, *p* = 4.40E-03) and hsa-miR-31-5p (OR = 4.188, *p* = 5.09E-02) were related to DR. ATP5A1 and OXA1L were closely correlated with each other in DR.

***Conclusion*:** The hsa-miR-31-5p-ATP5A1 and hsa-let-7b-5p-OXA1L axes might play novel and important roles in the pathogenesis and development of DR.

## 1 INTRODUCTION

Diabetic retinopathy (DR) is a leading cause of visual loss and a microvasculature complication of diabetes mellitus (DM). The prevalence of DR is estimated to be 191.0 million by 2030 [1]. The Wisconsin Epidemiologic Study of Diabetic Retinopathy showed that is a decline in the annual incidence of DR among patients with type 1 DM, but not in type 2 DM [1]. Also, the prevalence of any DR and proliferative DR in type 1 DM was higher than those in patients with type 2 DM [2]. However, the treatment methods for late-stage DR are not very effective and the pathogenesis of DR is still unclear.

The prevalence of DR is associated with multiple risk factors, including insulin use, hypertension, hyperglycemia, duration of diabetes, pregnancy, age, race, and blood pressure [1-4]. Also, the infection of coronavirus disease 2019 (COVID-19) impacts the monitoring and treatment for DR [5,6]. Potential diagnostic biomarkers, including oxidative stress biomarkers and inflammatory cytokines, had high accu-racies for DR [7, 8]. Biomarker discovery based on the integrated bioinformatics analysis of genomics has provided a lot of information on many aspects of medicine, including the diagnosis, development, and drug discovery of diseases [9, 10]. New diagnostic biomarkers, including miR-211, let-7a-5p, miR-1281, and miR-1179 have high accuracies in diagnosing DR [11-14]. Liu *et al*. [12] showed that miR-211 was up-regulated in DR when compared with controls, and its expression had high accuracy in diagnosing DR (area under curve, AUC=0.864). Also, a genome-wide association study by Meng *et al*. [15] suggested that the single nucleotide polymorphisms (rs10765219 and rs11018670) in the NADPH oxidase 4 gene were associated with the severity of DR in type 2 DM. These risk factors and biomarkers are helpful in stratifying the risk of retinopathy [1-4]. Therefore, the exploration of new diagnostic biomarkers DR is still necessary and might provide novel and important information for the diagnosis or treatment of DR.

The aim of this study was to identify differentially expressed genes (DEGs) in DR and the potential biomarkers that might provide additional information for the diagnosis of DR. Integrated bioinformatics and statistical analyses were performed to screen DEGs, DR-associated genes, and miRNAs. The study might provide a reference to the pathogenesis, diagnosis, or therapy of DR.

## 2 MATERIALS AND METHODS

### 2.1 Microarray Dataset Collection

DR microarray datasets GSE53257 (gene), GSE160306 (gene), and GSE160308 (miRNAs) were downloaded from the public functional genomics data repository Gene Expression Omnibus (GEO, https://www.ncbi.nlm.nih.gov/geo/). The GSE53257 dataset (GPL18056 platform, Homo sapiens Custom Human 8x15k Array designed by Genotypic Technology Private Limited (AMADID: 045815) included 16 neural retina samples collected from healthy donors (n = 5), donors with DR (n = 6), and donors with diabetes (n = 5). The GSE160306 and GSE160308 datasets (GPL20301, Illumina HiSeq 4000 (Homo sapiens) each included 79 samples, including 20 healthy controls and 59 DR donors (including 23 non-proliferative DRs and five proliferative DRs). The GSE53257 was used to screen differentially expressed genes (DEGs). The GSE160306 dataset was used to validate the expression profiles of the DR-associated genes. The GSE160308 dataset was used to screen out miRNAs that were differentially expressed between samples collected from DR donors and healthy donors and validate the miRNAs targeting DR-associated genes.

### 2.2 Screening of DEGs in DR retinal samples

The GEO2R tool provided by the GEO was used to screen the genes that were differentially expressed between the DR and control groups in the GSE53257 dataset. The criteria for selecting DEGs were set as |log(fold change)| ≥ 0.263, *p* < 0.05, and false discovery rate <0.1 to increase the number of DEGs and potential targets.

### 2.3 Functional enrichment analysis

The biological functions of the DEGs were analyzed in The Database for Annotation, Visualization, and Integrated Discovery (DAVID) online tool (version 6.8; https://david.ncifcrf.gov/). DAVID provides a comprehensive set of functional annotation tools to understand the biological meaning behind the DEGs across the DR and control samples. The biological themes, including the Gene Ontology (GO) functional terms and Kyoto Encyclopedia of Genes and Genomes (KEGG) pathways, associated with DEGs were identified with the cutoffs of hit ≥2 and *p* < 0.05.

### 2.4 Construction of the protein-protein interaction (PPI) network

The interaction pairs among DEGs were identified from the STRING online search tool (version 11.5; https://string-db.org/). The PPI pairs with a score of greater than 0.4 were retained and used for the construction of the PPI network. The Cytoscape software (version 3.8.0; https://apps.cytoscape.org/) was employed to construct the PPI network.

### 2.5 Identification of miRNAs of the DEGs

The miRNAs targeting DR-associated DEGs were identified from the miRTarbase, starBase, and TargetScan 7.2 (score >90) databases. The miRNA-mRNA pairs identified from at least two out of the three databases were retained and used to construct the miRNA-mRNA regulatory network, using the Cytoscape software.

### 2.6 Statistical analysis

The expression profiles of the key candidate genes and miRNAs in the GSE160306 and GSE160308 datasets were downloaded. The differences in the expression profiles of candidates across groups were analyzed using the non-parametric Kruskal-Wallis H test, with Dunn’s test for the correction. Also, the association of genes/miRNAs with DR was analyzed using the logistics regression analysis. The correlations between gene and miRNA expression levels in the DR samples were analyzed using the Spearman correlation coefficient analysis. The 95% confident interval (CI) and odds ratio (OR) was calculated for statistical analyses. For all statistical analyses, *p* < 0.05 was set as the significant threshold.

## 3 RESULTS

### 3.1 DEGs screening

**Fig. (1):**
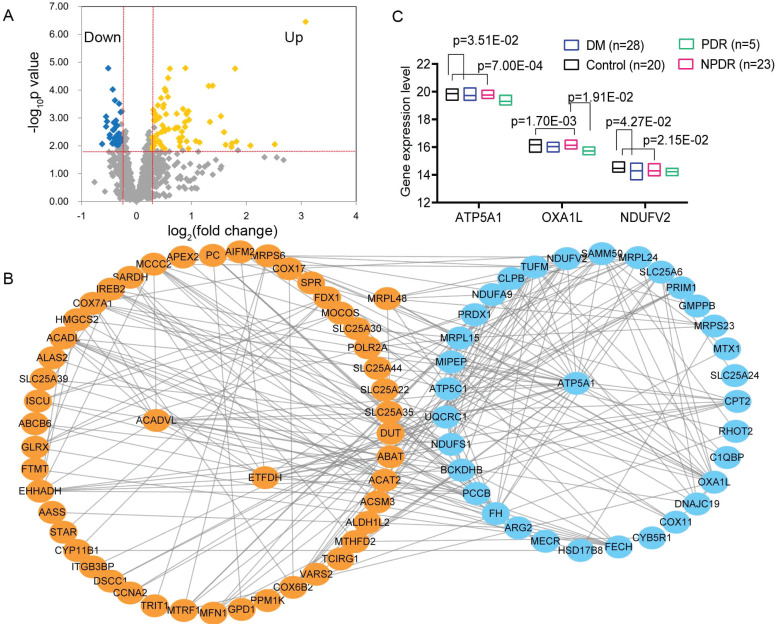
Screening of differentially expressed genes and hub genes. (**A**) The volcanic fog showing the differentially expressed genes in the GSE53257 dataset. (**B**) The protein-protein interaction network of the differentially expressed genes in diabetic retinopathy (DR). Up- and down-regulated genes are indicated by orange and blue nodes, respectively. Interactions are indicated by gray lines. (**C**) The different expression profiles of three hub genes between DR and control samples in the GSE160306 dataset. The expression profiles of three genes in the non-proliferative DR (NPDR) and proliferative DR (PDR) samples in the GSE160306 dataset. Differences were analysed using the non-parametric Kruskal-Wallis H test (Dunn’s corrections).

A total of 117 DEGs were identified from the GSE53257 dataset (Fig. **1A** and Table **S1**), of which 78 and 39 DEGs were of up-regulation and down-regulation in the neural retina from donors with a medical history of diabetes and signs of DR when compared with the control donors in the GSE53257 dataset (Table **S1**).

### 3.2 Functional annotations

Functional enrichment analysis showed that the 117 common DEGs were focused on 29 GO biological processes, including “GO:0006879:cellular iron ion homeostasis”, “GO:0055085: transmembrane transport”, “GO:0032543:mitochondrial translation”, and “GO:0009058: biosynthetic process” (Table **1** and Table **S2**), and 21 KEGG pathways, including “hsa01100:Metabolic pathways”, “hsa00190:Oxidative phosphorylation”, “hsa01212:Fatty acid metabolism”, and “hsa01130:Biosynthesis of antibiotics” (Table **1** and Table **S2**). Moreover, we found that 71 DEGs (62.28%) were the subunits or components of the mitochondrion and were cellular components of “GO:0005743: mitochondrial inner membrane”, “GO:0005746: mitochondrial respiratory chain”, and “GO:0031966: mitochondrial membrane”, with the molecular functions of “GO:0005515:protein binding”, “GO:0009055: electron carrier activity”, and “GO:0016491: oxidoreductase activity” (Table **1** and Table **S2**).

### 3.3 The PPI network

The PPI analysis of those DEGs generated a network consisted of 82 nodes and 254 lines (interaction pairs; Fig. **1B**), including 33 and 49 DEGs that were of down-regulation and up-regulation, respectively. The top 20 nodes with high interaction degrees included ATP5A1 (ATP synthase, H+ transporting, mitochondrial F1 complex, alpha subunit 1, cardiac muscle; degree =26), ATP5C1 (degree = 20), OXA1L (OXA1L, mitochondrial inner membrane protein; degree = 17), electron transfer flavoprotein dehydrogenase (ETFDH, degree = 12), and NADH dehydrogenase (ubiquinone) flavoprotein 2 (NDUFV2, degree = 10). Also, the ATP5A1 (down) interacted with OXA1L (down), ATP5C1 (down), NDUFV2 (down), and ETFDH (up; Fig. **1B**).

### 3.4 DEGs Associated with DR

Among the top 20 DEGs in the PPI network, only three genes were differentially expressed between the DR and control samples in the GSE160306 dataset (Fig. **1C**). The Kruskal-Wallis H test showed that the ATP5A1, OXA1L, and NDUFV2 genes were down-regulated in the retinal samples from patients with DM and proliferative DR compared with the control samples in the GSE160306 dataset (Fig. **1C**). Also, only OXA1L was differentially expressed between the samples from the non-proliferative and proliferative DR samples (Fig. **1C**).

Univariate logistics regression analysis showed that ATP5A1, OXA1L, and NDUFV2 were associated with DR occurrence in the GSE160306 dataset (Table **2**), as high expression levels of ATP5A1 (OR = 0.007, 95% CI 0.000-0.368, *p* =1.40E-02), NDUFV2 (OR = 0.003, 95% CI 0.000-0.199, *p* = 6.40E-03), and OXA1L (OR = 0.093, 95% CI 0.005-0.680, *p* = 3.08E-02) were associated with low risk of DR. These results showed that these DEGs might play crucial roles in the pathogenesis of DR.

### 3.5 Screening of miRNAs of DEGs

**Fig. (2):**
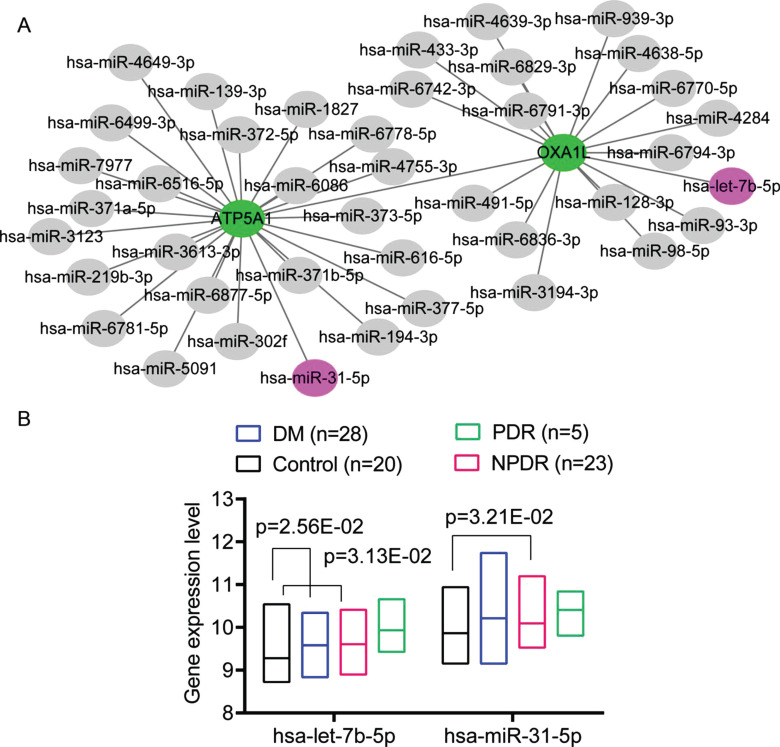
The hub miRNA-mRNA regulatory network in diabetic retinopathy (DR). (**A**) the hub miRNA-mRNA regulatory network. Up- and down-regulated genes are indicated by purple and green nodes. Interactions are indicated by gray lines. Grey nodes indicate miRNAs with unknown changes in the GSE160308 dataset. (**B**) the expression profiles of two hub miRNAs in the GSE160308 dataset. DR, diabetic retinopathy. Differences were analysed using the non-parametric Kruskal-Wallis H test (Dunn’s corrections).

We identified that the ATP5A1 and OXA1L genes were targeted by 25 and 17 miRNAs, respectively, based on the miRNA-mRNA screening in the databases of miRTarbase, starBase, and TargetScan 7.2. There was no miRNA targeting NDUFV2. The miRNA-mRNA regulatory network is shown in Fig. (**2A**), in which 44 miRNAs and two genes were included. Among the miRNAs targeting ATP5A1 and OXA1L, two were differentially expressed between the DR and control samples in the GSE160308 dataset (Fig. **2B**). Accordingly, the network included two DEGs and 44 miRNAs, including two up-regulated miRNAs in DR in the GSE160308 dataset (hsa-let-7b-5p and hsa-miR-31-5p); Fig. (**2A**). OXA1L was regulated by hsa-let-7b-5p and ATP5A1 was by hsa-miR-31-5p (Fig. **2A**).

### 3.6 miRNAs Associated with DR

Univariate logistics regression analysis showed that miRNAs hsa-let-7b-5p and hsa-miR-31-5p were associated with DR occurrence in the GSE160306 dataset (Table **2**). Logistics regression analysis showed that high expression levels of hsa-let-7b-5p (OR = 26.071, 95% CI 2.764-245.954, *p* = 4.40E-03) and hsa-miR-31-5p (OR = 4.188, 95% CI 0.995-17.636, *p* = 5.09E-02) were associated with a high risk of DR (Table **2**). These results showed that hsa-miR-31-5p and hsa-let-7b-5p might play crucial roles in the pathogenesis of DR.

### 3.7 Correlation between miRNAs and DEGs

**Fig. (3):**
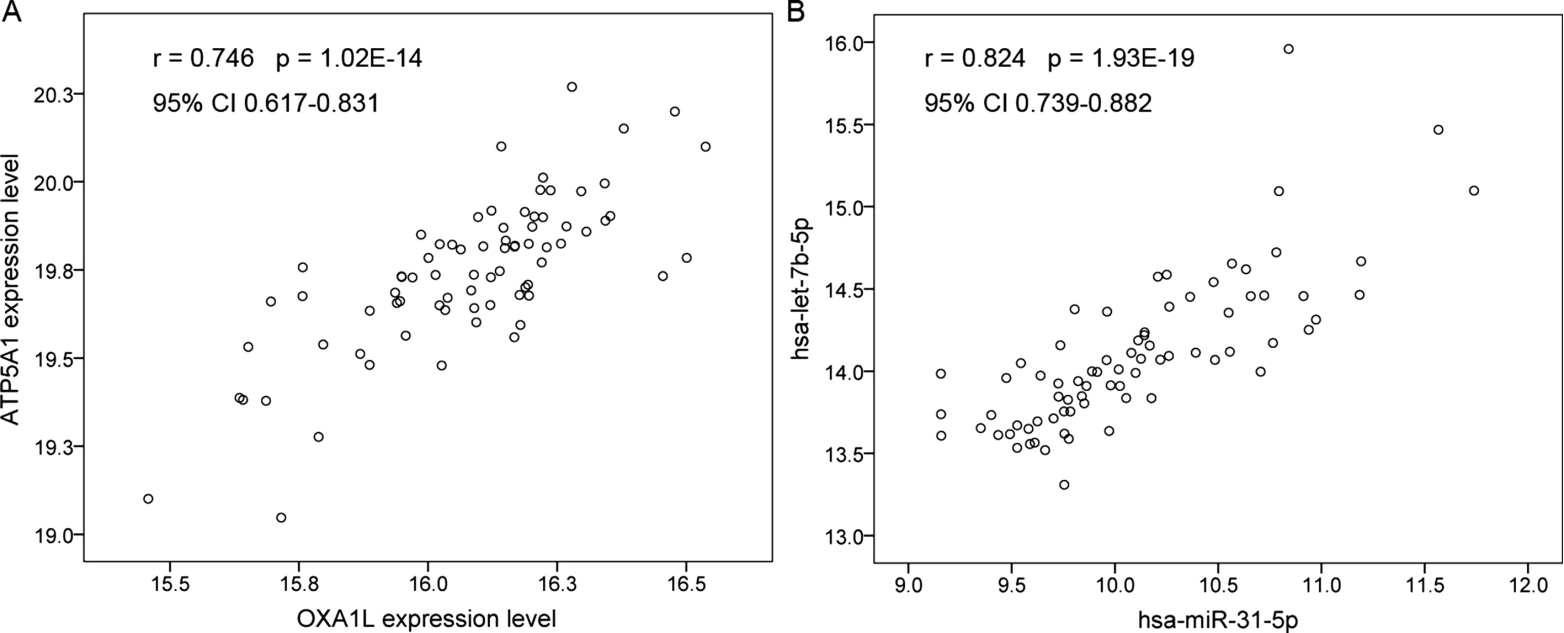
The correlation between miRNAs and genes. (**A**) the correlation between the ATP5A1 and OXA1L gene expression profiles. (**B**) the correlation between the let-7b-5p and miR-31-5p levels. The correlation was analysed using the Spearman correlation coefficiant analysis.

The correlations between miRNAs and DEGs in the GSE160308 and GSE160306 datasets were analyzed using the Spearman correlation coefficients. We found that the expression levels of the ATP5A1 and OXA1L genes were closely correlated in the GSE160306 dataset (r =0.746, *p* = 1.02E-14; Fig. **3A**), and hsa-let-7b-5p was correlated with hsa-miR-31-5p (r = 0.824, *p* = 1.93E-19; Fig. **3B**) in the GSE160308 dataset, respectively. There were no significant correlations between miRNAs and mRNAs.

## 4 DISCUSSION

The use of bioinformatics analysis of genomics in exploring biomarkers in diseases provides novel and important references in disease diagnosis, prognostic prediction, and clinical therapy [11-14]. We used integrated bioinformatics analysis and found that three DEGs, including ATP5A1, NDUFV2, and OXA1L, and two miRNAs, including hsa-let-7b-5p and hsa-miR-31-5p, associated with the occurrence of DR significantly. We showed that identified that hsa-miR-31-5p-ATP5A1 and hsa-let-7b-5p -OXA1L regulatory axes may play novel and important roles in the pathogenesis and development of DR.

Three hub genes, including ATP5A1, NDUFV2, and OXA1L, were mainly focused on the biological process terms related to “GO:0055085: transmembrane transport” and “GO:0032543:mitochondrial translation”. NDUFV2 is a subunit of mitochondrial complex I and its dysregulation is associated with animal longevity and a variety of diseases [16, 17]. It participates in conditions with an association with mitochondrial dysfunction [16]. The ATP5A1 gene, also named ATP5F1A, encodes a subunit of mitochondrial ATP synthase that participates in and catalyzes ATP synthesis, and coupled proton transport in the mitochondrion. Mitochondrial ATP production is the main energy source for intracellular metabolic pathways. The low level of ATP5A1 is associated with microsatellite instability and the development of human diseases [18-20]. Studies have shown that ATP5A1 mediates cellular apoptosis, innate immune response, tumorigenesis, and angiogenesis [19, 20]. The expression level of ATP5A1 was higher in normal kidneys than in clear cell renal cell carcinoma tissues [19, 21], but was higher in glioblastoma tumor cells when compared with normal brain blood vessels [22]. Also, the complex V ATP5A1 defect interferes with the stability of the complex and causes fatal neonatal mitochondrial encephalopathy [18]. These results suggested that the ATP5A1 gene played a crucial role in DR pathogenesis by regulating mitochondrial ATP production.

There is less information showing the association of OXA1L with human disease. OXA1L encodes an evolutionarily conserved protein localized to the mitochondrial inner membrane. This protein is involved in the assembly of the cytochrome c oxidase and ATPase complexes of the mitochondrial respiratory chain [23]. Also, it is important for the stability of the mitoribosome [24]. The variants or polymorphisms in the OXA1L gene were associated with asthma and allergy by regulating the biogenesis and mitochondrial oxidative phosphorylation [25]. Our present study showed that the ATP5A1 and OXA1L genes were down-regulated in DR, and were decreased in proliferative DR compared with non-proliferative DR. The two genes had a close correlation (r=0.746). The downregulation of the ATP5A1 and OXA1L genes in DR and proliferative DR might indicate that mitochondrial ATP synthesis was associated with the pathogenesis and development of DR.

Among the DR-associated miRNAs, we identified that hsa-miR-31-5p negatively regulated ATP5A1 and hsa-let-7b-5p negatively regulated OXA1L. The two miRNAs were up-regulated in DR when compared with controls. Evidence showed that the two genes were associated with a variety of biological processes, including cell proliferation and oxaliplatin resistance, by regulating their target genes [26-30]. Li *et al*. [30] showed that hsa-let-7b-5p is a mitochondrial miRNA, and its expression enhanced mitochondrial translation, reduced the production of reactive oxygen species, decreased lipid deposition, and finally rescued diabetic cardiomyopathy. These data showed that let-7b-5p might have a protective role in diabetic cardiomyopathy. Our study showed that hsa-miR-31-5p and hsa-let-7b-5p, negatively regulated ATP5A1 or OXA1L, and were associated with DR. The present study revealed the significance of the hsa-miR-31-5p-ATP5A1 and hsa-let-7b-5p-OXA1L axes in the pathogenesis and development of DR.

## CONCLUSION

To sum up, we identified three DR-associated genes (ATP5A1, NDUFV2, and OXA1L) and two DR-associated miRNAs (hsa-let-7b-5p and hsa-miR-31-5p) using integrated bioinformatics analysis. Logistics regression analysis showed that these genes and miRNAs were associated with DR. The hsa-miR-31-5p-ATP5A1 and hsa-let-7b-5p-OXA1L axes were of great interest in the pathogenesis and development of DR by regulating mitochondrial ATP production. However, experiments and clinical trials verifying the hypotheses might be of great value in DR.

## AUTHORS' CONTRIBUTIONS

The conception and design of the research were conducted by Weihai Xu and Zhilan Yuan. Acquisition, analysis, and interpretation of data were collected by Weihai Xu, Ya Liang, and Ying Zhuang. Drafting the manuscript was done by Weihai Xu. Revision of the manuscript for important intellectual content was done by Ya Liang, Ying Zhuang, and Zhilan Yuan. All authors have read and approved the manuscript.

## Figures and Tables

**Table 1:** The top 10 biological processes and pathways associated with the differentially expressed genes in the GSE53257 datasets.

**-Category**	**Count**	***p* Value**	**Genes**
**GO biological process terms**	-	-	-
GO:0006879:cellular iron ion homeostasis	5	1.89E-04	ALAS2, FTMT, ABCB6, IREB2, ISCU
GO:0055085:transmembrane transport	8	1.96E-04	SLC25A27, SLC25A39, ABCB6, SLC25A19, SLC25A40, SLC25A22, SLC25A44, SLC25A24
GO:0009060:aerobic respiration	5	3.91E-04	NDUFA9, OXA1L, UQCRC1, NDUFS1, NDUFV2
GO:0033539:fatty acid beta-oxidation using acyl-CoA dehydrogenase	3	1.28E-03	ACADVL, ACADL, ETFDH
GO:0032543:mitochondrial translation	5	1.29E-03	MRPS23, MRPL15, MRPL48, MRPL24, MRPS6
GO:0006635:fatty acid beta-oxidation	4	1.69E-03	CPT2, EHHADH, ALDH1L2, ACAT2
GO:0006633:fatty acid biosynthetic process	4	2.25E-03	MECR, ACSM3, PCCB, HSD17B8
GO:0009083:branched-chain amino acid catabolic process	3	2.74E-03	MCCC2, BCKDHB, SLC25A44
GO:0045471:response to ethanol	5	4.55E-03	STAR, FECH, ABAT, HMGCS2, TUFM
GO:0006783:heme biosynthetic process	3	6.66E-03	ALAS2, SLC25A39, FECH
**KEGG pathways**	-	-	-
hsa01100:Metabolic pathways	38	1.98E-12	ALAS2, FH, ACADVL, ACSM3, FECH, COX17, ABAT, TCIRG1, COX7A1, ALDH1L2, HSD17B8, ACAT2, GMPPB, ACADL, SPR, COX11, HMGCS2, NDUFV2, AASS, MCCC2, NDUFA9, ARG2, DUT, MECR, GLYCTK, BCKDHB, MOCOS, ACYP2, COX6B2, TRIT1, PC, MTHFD2, EHHADH, CYP11B1, PCCB, UQCRC1, SARDH, NDUFS1
hsa00280:Valine, leucine and isoleucine degradation	7	1.53E-06	MCCC2, EHHADH, BCKDHB, PCCB, ABAT, HMGCS2, ACAT2
hsa01212:Fatty acid metabolism	7	4.30E-06	ACADVL, MECR, CPT2, ACADL, EHHADH, HSD17B8, ACAT2
hsa00190:Oxidative phosphorylation	9	7.47E-06	NDUFA9, COX17, UQCRC1, COX11, NDUFS1, TCIRG1, NDUFV2, COX6B2, COX7A1
hsa00650:Butanoate metabolism	5	4.85E-05	ACSM3, EHHADH, ABAT, HMGCS2, ACAT2
hsa00071:Fatty acid degradation	5	3.11E-04	ACADVL, CPT2, ACADL, EHHADH, ACAT2
hsa04714:Thermogenesis	9	3.67E-04	NDUFA9, CPT2, COX17, UQCRC1, COX11, NDUFS1, NDUFV2, COX6B2, COX7A1
hsa05415:Diabetic cardiomyopathy	8	8.75E-04	NDUFA9, CPT2, UQCRC1, NDUFS1, NDUFV2, COX6B2, COX7A1, SLC25A6
hsa00640:Propanoate metabolism	4	1.83E-03	EHHADH, BCKDHB, PCCB, ABAT
hsa05012:Parkinson disease	8	4.08E-03	NDUFA9, MFN1, UQCRC1, NDUFS1, NDUFV2, COX6B2, COX7A1, SLC25A6

**Table 2:** Univariate logistics regression analysis of the genes and miRNAs associated with diabetic retinopathy in the GSE160306 dataset.

**Gene Symbol**	**β**	***p* Value**	**OR**	**95% CI**
Gender	0.693	2.44e-01	2.000	0.623-6.421
Age	-0.022	4.91e-01	0.978	0.918-1.042
ATP5A1	-4.569	6.02E-03	0.010	0.000-0.270
ETFDH	-6.098	1.94E-03	0.002	0.000-0.106
OXA1L	-3.032	3.67E-02	0.048	0.003-0.829
NDUFV2	-5.736	6.40E-03	0.003	0.000-0.199
hsa-let-7b-5p	3.100	2.52E-03	22.188	2.969-165.804
hsa-miR-128-3p	-1.652	5.29E-02	0.192	0.036-1.021
hsa-miR-31-5p	1.265	3.32E-02	3.542	1.106-11.341
hsa-miR-98-5p	1.614	1.06E-02	5.020	1.456-17.316

**Abbreviations:** OR, odds ratio. CI, confident interval.

## Data Availability

The gene/miRNA expression datasets are available from the National Center for Biotechnology Information Gene Expression Omnibus (http://www.ncbi.nlm.nih.gov/geo) with the accession numbers of GSE53257, GSE60436, GSE160 306, and GSE160308.

## LIST OF ABBREVIATIONS

DAVID: Database for Annotation, Visualization and Integrated Discovery
DEGs: Differentially Expressed Genes
DM: Diabetes Mellitus
DR: Diabetic Retinopathy
GEO: Gene Expression Omnibus
GO: Gene Ontology
KEGG: Kyoto Encyclopedia of Genes and Genomes

## SUPPLEMENTARY MATERIALS

Supplementary material is available on the publisher’s website along with the published article. Table **S1**. The list of the 68 common differentially ex-pressed genes with co-expression profiles in the GSE53257 and GSE30436 datasets. Table **S2**. The functional annotation of the 68 common dif-ferentially expressed genes in the GSE53257 and GSE60436 datasets.
