# Supplementary material for: Identification of miRNA-mRNA Regulatory Networks Associated with Diabetic Retinopathy using Bioinformatics Analysis
Source: Endocr Metab Immune Disord Drug Targets. 2023 Oct 11;23(13):1628–36. doi: 10.2174/1871530323666230419081351 (PMC10661965; doi:10.2174/1871530323666230419081351)
Supplement: Supplementary file 1 — Supplementary material is available on the publisher’s website along with the published article. Table S1. The list of the 68 common differentially ex-pressed genes with co-expression profiles in the GSE53257 and GSE30436 datasets. Table S2. The functional annotation of the 68 common dif-ferentially expressed genes in the GSE53257 and GSE60436 datasets. [file EMIDDT-23-1628_SD1.pdf]

Supplementary Material

Identification of miRNA-mRNA Regulatory Networks associated with Diabetic Retinopathy using Bioinformatics Analysis

Weihai Xu[1,2], Ya Liang[1], Ying Zhuang[3] and Zhilan Yuan[1,*]

[1]Department of Ophthalmology, the First Affiliated Hospital of Nanjing Medical University, Nanjing, 210029, China; [2]Department of Ophthalmology, the Binhai County People's Hospital, Yancheng, China, 224500; [3]Department of Stomatology, the Binhai County People's Hospital, Yancheng, 224500, China,

Table S1. The 114 differentially expressed genes in the GSE53257 and GSE30436 datasets.

Gene	logFC	FDR	*P* Value
ACSM3	3.08	3.97E-04	3.55E-07
ARMC4	2.52	8.80E-02	8.96E-03
ALDH1L2	2.08	9.00E-02	9.91E-03
COX17	1.82	7.98E-02	7.13E-03
DSCC1	1.8	3.86E-03	1.71E-05
SLC25A34	1.74	8.42E-02	8.27E-03
FTMT	1.73	9.19E-02	1.11E-02
HMGCS2	1.62	8.87E-02	9.26E-03
RHOH	1.6	5.68E-02	3.27E-03
CYB5R2	1.54	3.25E-02	8.74E-04
SARDH	1.39	1.01E-02	7.03E-05
EHHADH	1.34	3.81E-02	1.39E-03
VISA	1.32	1.01E-02	7.22E-05
MTHFD2	1.15	8.17E-02	7.59E-03
ACADL	1.08	7.98E-02	6.85E-03
SLC25A30	0.96	3.62E-02	1.17E-03
STAR	0.95	9.83E-02	1.28E-02
PNKD	0.95	2.86E-02	5.76E-04
ALAS2	0.92	2.86E-02	6.31E-04
GLRX	0.92	7.98E-02	6.96E-03
tcag7.1260	0.91	2.86E-02	7.41E-04
LMO2	0.89	3.86E-03	1.64E-05
TCIRG1	0.89	4.83E-02	2.11E-03
COX6B2	0.88	3.82E-02	1.44E-03
MOCOS	0.85	2.83E-02	4.81E-04
GPD1	0.83	7.13E-02	4.91E-03
CYP11B1	0.83	5.68E-02	3.14E-03
SPR	0.8	7.98E-02	7.20E-03
COX7A1	0.79	9.02E-02	1.05E-02
AIFM2	0.78	7.25E-02	5.44E-03

SLC25A44	0.76	4.90E-02	2.24E-03
KRT5	0.74	2.86E-02	7.13E-04
CCNA2	0.72	4.90E-02	2.28E-03
C14orf68	0.7	5.10E-02	2.46E-03
TSHZ3	0.61	3.86E-03	1.72E-05
SLC25A35	0.59	5.12E-02	2.56E-03
SLC25A19	0.58	6.97E-03	3.74E-05
PPM1K	0.57	1.65E-02	1.77E-04
PC	0.54	2.86E-02	7.40E-04
ETFDH	0.53	1.12E-02	1.10E-04
ACAT2	0.52	1.21E-01	1.85E-02
POLR2A	0.52	5.65E-02	2.99E-03
ACYP2	0.52	1.07E-02	9.06E-05
ACAT2	0.51	9.83E-02	1.28E-02
GLYCTK	0.51	5.10E-02	2.51E-03
SUPV3L1	0.5	2.34E-02	3.34E-04
NIPSNAP3B	0.49	2.68E-02	4.32E-04
IREB2	0.47	4.58E-02	1.85E-03
AASS	0.47	9.02E-02	1.06E-02
VARS2	0.46	5.68E-02	3.21E-03
SLC25A22	0.45	8.90E-02	9.46E-03
SLMO1	0.45	1.67E-02	1.94E-04
ABCB6	0.42	7.98E-02	7.19E-03
EEFSEC	0.42	3.27E-02	9.64E-04
ACADVL	0.41	8.27E-02	7.84E-03
PPIP5K2	0.4	3.39E-02	1.03E-03
MTERF	0.4	7.98E-02	6.87E-03
MRPS6	0.39	2.36E-02	3.58E-04
MOSC2	0.38	6.40E-02	3.90E-03
NIPSNAP3A	0.37	3.27E-02	9.34E-04
MCCC2	0.35	7.02E-02	4.70E-03
SLC25A40	0.35	3.62E-02	1.24E-03
MFN1	0.34	2.86E-02	7.07E-04
FDX1	0.33	3.62E-02	1.14E-03
SLC25A27	0.33	3.62E-02	1.26E-03
ACYP2	0.33	4.90E-02	2.23E-03
ABAT	0.33	8.27E-02	7.98E-03
MTRF1	0.32	7.98E-02	7.09E-03
ISCU	0.32	2.86E-02	7.14E-04
SLC25A39	0.32	9.00E-02	9.96E-03
C12orf62	0.31	2.86E-02	5.20E-04
MRPL48	0.31	9.02E-02	1.05E-02

ITGB3BP	0.3	9.07E-02	1.07E-02
DUT	0.29	9.83E-02	1.28E-02
TRIT1	0.29	7.15E-02	5.09E-03
COX2	0.29	7.15E-02	5.06E-03
APEX2	0.27	9.21E-02	1.13E-02
RILP	0.27	7.55E-02	5.93E-03
ARG2	-0.27	6.85E-02	4.41E-03
TUFM	-0.28	4.33E-02	1.70E-03
UQCRC1	-0.28	9.80E-02	1.23E-02
PRDX1	-0.29	2.86E-02	6.25E-04
MTX1	-0.29	7.15E-02	5.17E-03
C1QBP	-0.29	7.98E-02	6.45E-03
CYB5R1	-0.3	8.10E-02	7.38E-03
NDUFS1	-0.31	7.27E-02	5.58E-03
CPT2	-0.32	2.31E-02	3.10E-04
MRPL24	-0.32	6.16E-02	3.64E-03
OXA1L	-0.33	8.91E-02	9.56E-03
DNAJC19	-0.34	6.40E-02	3.96E-03
ATP5A1	-0.35	8.29E-02	8.07E-03
SLC25A6	-0.35	4.58E-02	1.91E-03
CLPB	-0.35	8.87E-02	9.12E-03
C9orf46	-0.36	8.17E-02	7.55E-03
NDUFV2	-0.36	7.02E-02	4.60E-03
TUFM	-0.36	9.02E-02	1.02E-02
GMPPB	-0.36	4.58E-02	1.92E-03
TUFM	-0.37	3.62E-02	1.25E-03
PCCB	-0.38	8.56E-02	8.57E-03
FH	-0.38	7.55E-02	5.89E-03
ATP5C1	-0.39	1.89E-02	2.37E-04
PRIM1	-0.4	5.10E-02	2.47E-03
RHOT2	-0.41	7.25E-02	5.31E-03
MRPL15	-0.41	3.82E-02	1.47E-03
BCKDHB	-0.41	5.22E-02	2.68E-03
NT5C3	-0.41	7.98E-02	7.16E-03
FECH	-0.42	8.24E-02	7.73E-03
HINT2	-0.42	5.22E-02	2.71E-03
NDUFA9	-0.43	1.07E-02	9.59E-05
SLC25A24	-0.44	8.50E-02	8.43E-03
TWINKLE	-0.48	6.40E-02	4.04E-03
SAMM50	-0.48	7.15E-02	5.18E-03
MIPEP	-0.51	3.86E-03	1.66E-05
MECR	-0.53	3.77E-02	1.35E-03

HSD17B8	-0.55	4.83E-02	2.07E-03
MRPS23	-0.55	3.25E-02	8.99E-04
COX11	-0.62	8.59E-02	8.68E-03

Abbreviations: FC, fold change. FDR, false discovery rate.

Table S2. The functional items associated with the differentially expressed genes in the GSE53257 datasets.

Category	Count	*P* Value	Genes
**GO biological process terms**			
GO:0006879:cellular iron ion homeostasis	5	1.89E-04	ALAS2, FTMT, ABCB6, IREB2, ISCU
GO:0055085:transmembrane transport	8	1.96E-04	SLC25A27, SLC25A39, ABCB6, SLC25A19, SLC25A40, SLC25A22, SLC25A44, SLC25A24
GO:0009060:aerobic respiration	5	3.91E-04	NDUFA9, OXA1L, UQCRC1, NDUFS1, NDUFV2
GO:0033539:fatty acid beta-oxidation using acyl-CoA dehydrogenase	3	1.28E-03	ACADVL, ACADL, ETFDH
GO:0032543:mitochondrial translation	5	1.29E-03	MRPS23, MRPL15, MRPL48, MRPL24, MRPS6
GO:0006635:fatty acid beta-oxidation	4	1.69E-03	CPT2, EHHADH, ALDH1L2, ACAT2
GO:0006633:fatty acid biosynthetic process	4	2.25E-03	MECR, ACSM3, PCCB, HSD17B8
GO:0009083:branched-chain amino acid catabolic process	3	2.74E-03	MCCC2, BCKDHB, SLC25A44
GO:0045471:response to ethanol	5	4.55E-03	STAR, FECH, ABAT, HMGCS2, TUFM
GO:0006783:heme biosynthetic process	3	6.66E-03	ALAS2, SLC25A39, FECH
GO:0008203:cholesterol metabolic process	4	7.95E-03	STAR, FECH, CYP11B1, FDX1
GO:0006694:steroid biosynthetic process	3	9.50E-03	HINT2, STAR, FDX1
GO:0009058:biosynthetic process	3	1.08E-02	GMPPB, ALAS2, ALDH1L2
GO:0006810:transport	3	1.08E-02	SLC25A39, SLC25A40, SLC25A22
GO:1904234:positive regulation of aconitate hydratase activity	2	1.47E-02	FTMT, ISCU
GO:0055072:iron ion homeostasis	3	1.98E-02	FECH, IREB2, ISCU
GO:0006091:generation of precursor metabolites and energy	3	2.24E-02	FECH, COX17, COX7A1
GO:0006120:mitochondrial electron transport, NADH to ubiquinone	3	2.24E-02	NDUFA9, NDUFS1, NDUFV2
GO:0006778:porphyrin-containing compound metabolic process	2	2.43E-02	ALAS2, ABCB6
GO:1902600:hydrogen ion transmembrane transport	4	3.06E-02	UQCRC1, TCIRG1, COX7A1, SLC25A22
GO:0071373:cellular response to luteinizing hormone stimulus	2	3.39E-02	CCNA2, STAR
GO:0022900:electron transport chain	3	3.41E-02	FDX1, ETFDH, COX11
GO:0071320:cellular response to cAMP	3	3.52E-02	STAR, GPD1, FDX1
GO:0032981:mitochondrial respiratory chain complex I assembly	3	3.84E-02	NDUFA9, OXA1L, NDUFS1
GO:0042776:mitochondrial ATP synthesis coupled proton transport	3	4.07E-02	NDUFA9, NDUFS1, NDUFV2
GO:0046322:negative regulation of fatty acid oxidation	2	4.34E-02	ACADVL, ACADL

GO:0006782:protoporphyrinogen IX biosynthetic process	2	4.81E-02	ALAS2, IREB2
GO:0034101:erythrocyte homeostasis	2	4.81E-02	PRDX1, IREB2
GO:0006879:cellular iron ion homeostasis	5	1.89E-04	ALAS2, FTMT, ABCB6, IREB2, ISCU
**GO molecular function terms**			
GO:0051537:2 iron, 2 sulfur cluster binding	5	8.83E-06	FECH, FDX1, NDUFS1, NDUFV2, ISCU
GO:0009055:electron carrier activity	6	2.43E-05	FDX1, ETFDH, COX11, NDUFS1, NDUFV2, COX7A1
GO:0051536:iron-sulfur cluster binding	4	1.90E-04	IREB2, ABAT, NDUFS1, ISCU
GO:0051539:4 iron, 4 sulfur cluster binding	4	1.60E-03	IREB2, ETFDH, NDUFS1, ISCU
GO:0050660:flavin adenine dinucleotide binding	4	4.58E-03	ACADVL, ACADL, AIFM2, ETFDH
GO:0008198:ferrous iron binding	3	8.32E-03	FTMT, FECH, ISCU
GO:0004174:electron-transferring-flavoprotein dehydrogenase activity	2	9.81E-03	AIFM2, ETFDH
GO:0030350:iron-responsive element binding	2	1.47E-02	FECH, IREB2
GO:0008137:NADH dehydrogenase (ubiquinone) activity	3	1.90E-02	NDUFA9, NDUFS1, NDUFV2
GO:0004466:long-chain-acyl-CoA dehydrogenase activity	2	1.95E-02	ACADVL, ACADL
GO:0016874:ligase activity	3	1.98E-02	MCCC2, PC, PCCB
GO:0003824:catalytic activity	4	2.74E-02	ALAS2, EHHADH, BCKDHB, ABAT
GO:0016491:oxidoreductase activity	5	3.12E-02	CYB5R2, PRDX1, SARDH, ETFDH, NDUFV2
GO:0005506:iron ion binding	4	3.36E-02	FTMT, CYP11B1, FDX1, ISCU
GO:0003857:3-hydroxyacyl-CoA dehydrogenase activity	2	3.39E-02	EHHADH, HSD17B8
GO:0097177:mitochondrial ribosome binding	2	3.39E-02	OXA1L, C1QBP
GO:0030170:pyridoxal phosphate binding	3	3.42E-02	ALAS2, MOCOS, ABAT
GO:0004128:cytochrome-b5 reductase activity, acting on NAD(P)H	2	3.87E-02	CYB5R2, CYB5R1
GO:0005515:protein binding	71	4.80E-02	ACADVL, ALAS2, CLPB, DSCC1, TCIRG1, ALDH1L2, GMPPB, ACADL, CPT2, AIFM2, C1QBP, MCCC2, ARG2, LMO2, MRPS23, BCKDHB, RHOH, ACYP2, KRT5, MRPL48, COX6B2, MRPS6, TUFM, SUPV3L1, CCNA2, OXA1L, MTHFD2, EHHADH, GPD1, PCCB, UQCRC1, NDUFS1, ISCU, ITGB3BP, SLC25A6, FH, TSHZ3, FECH, PRIM1, IREB2, COX17, ETFDH, GLRX, MRPL15, PPM1K, COX7A1, HSD17B8, ACAT2, CYB5R2, CYB5R1, RHOT2, SAMM50, POLR2A, PRDX1, MFN1, COX11, VARS2, NDUFV2, RILP, DNAJC19, NDUFA9, DUT, NIPSNAP3A, MTX1, GLYCTK, MOCOS, APEX2, PNKD, PC, STAR, SLC25A30
GO:0005525:GTP binding	6	4.96E-02	GMPPB, EEFSEC, RHOT2, MFN1, RHOH, TUFM
**GO cellular component terms**			
GO:0005739:mitochondrion	71	5.52E-59	MIPEP, ACADVL, ALAS2, ACSM3, CLPB, ABAT, ALDH1L2, MTRF1, HINT2, ACADL, CPT2, AIFM2, C1QBP, SLC25A40, HMGCS2, SLC25A44, AASS, MCCC2, ARG2, MECR, MRPS23, BCKDHB, MRPL48, COX6B2, MRPS6, TUFM, SUPV3L1, OXA1L, MTHFD2, SLC25A19, FDX1, PCCB, UQCRC1, NDUFS1, ISCU, SLC25A6, FH, FECH, ABCB6, IREB2, GLRX, MRPL15, PPM1K, COX7A1, ACAT2, SLC25A27, CYB5R1, RHOT2, SAMM50, FTMT, MFN1, COX11, VARS2, NDUFV2, SLC25A24, NIPSNAP3B, DNAJC19, NDUFA9, DUT, NIPSNAP3A, GLYCTK, APEX2, MRPL24, TRIT1, PNKD, SLC25A39, PC, STAR, CYP11B1, SLC25A30, SARDH
GO:0005759:mitochondrial matrix	31	1.95E-28	MIPEP, ALAS2, FH, ACADVL, ACSM3, FECH, ETFDH, ABAT, PPM1K, ALDH1L2, HSD17B8, FTMT, ACADL, C1QBP, HMGCS2, AASS, MCCC2, NDUFA9, ARG2, MECR, BCKDHB, TRIT1, SUPV3L1, OXA1L, PC, MTHFD2, PCCB, FDX1, SARDH, NDUFS1, ISCU

GO:0005743:mitochondrial inner membrane	26	1.61E-19	ALAS2, FECH, ETFDH, MRPL15, SLC25A27, CPT2, COX11, SLC25A40, NDUFV2, SLC25A22, SLC25A24, DNAJC19, MRPS23, MRPL48, MRPS6, MRPL24, OXA1L, SLC25A39, SLC25A19, CYP11B1, SLC25A30, UQCRC1, NDUFS1, SLC25A34, SLC25A35, SLC25A6
GO:0031966:mitochondrial membrane	8	8.03E-06	NDUFA9, SLC25A27, OXA1L, ACADVL, ACADL, SLC25A30, ETFDH, SLC25A44
GO:0005741:mitochondrial outer membrane	8	4.97E-05	RHOT2, SAMM50, HINT2, MTX1, ABCB6, AIFM2, MFN1, TUFM
GO:0031305:integral component of mitochondrial inner membrane	4	9.50E-04	OXA1L, SLC25A19, ETFDH, COX11
GO:0031307:integral component of mitochondrial outer membrane	3	5.22E-03	RHOT2, ABCB6, MFN1
GO:0005758:mitochondrial intermembrane space	4	8.87E-03	STAR, CLPB, COX17, NDUFS1
GO:0042645:mitochondrial nucleoid	3	1.99E-02	SUPV3L1, ACADVL, TUFM
GO:0005747:mitochondrial respiratory chain complex I	3	2.33E-02	NDUFA9, NDUFS1, NDUFV2
GO:0005762:mitochondrial large ribosomal subunit	3	2.97E-02	MRPL15, MRPL48, MRPL24
GO:0030061:mitochondrial crista	2	4.62E-02	STAR, COX6B2
**KEGG pathways**			
hsa01100:Metabolic pathways	38	1.98E-12	ALAS2, FH, ACADVL, ACSM3, FECH, COX17, ABAT, TCIRG1, COX7A1, ALDH1L2, HSD17B8, ACAT2, GMPPB, ACADL, SPR, COX11, HMGCS2, NDUFV2, AASS, MCCC2, NDUFA9, ARG2, DUT, MECR, GLYCTK, BCKDHB, MOCOS, ACYP2, COX6B2, TRIT1, PC, MTHFD2, EHHADH, CYP11B1, PCCB, UQCRC1, SARDH, NDUFS1
hsa00280:Valine, leucine and isoleucine degradation	7	1.53E-06	MCCC2, EHHADH, BCKDHB, PCCB, ABAT, HMGCS2, ACAT2
hsa01212:Fatty acid metabolism	7	4.30E-06	ACADVL, MECR, CPT2, ACADL, EHHADH, HSD17B8, ACAT2
hsa00190:Oxidative phosphorylation	9	7.47E-06	NDUFA9, COX17, UQCRC1, COX11, NDUFS1, TCIRG1, NDUFV2, COX6B2, COX7A1
hsa00650:Butanoate metabolism	5	4.85E-05	ACSM3, EHHADH, ABAT, HMGCS2, ACAT2
hsa00071:Fatty acid degradation	5	3.11E-04	ACADVL, CPT2, ACADL, EHHADH, ACAT2
hsa04714:Thermogenesis	9	3.67E-04	NDUFA9, CPT2, COX17, UQCRC1, COX11, NDUFS1, NDUFV2, COX6B2, COX7A1
hsa05415:Diabetic cardiomyopathy	8	8.75E-04	NDUFA9, CPT2, UQCRC1, NDUFS1, NDUFV2, COX6B2, COX7A1, SLC25A6
hsa00640:Propanoate metabolism	4	1.83E-03	EHHADH, BCKDHB, PCCB, ABAT
hsa05012:Parkinson disease	8	4.08E-03	NDUFA9, MFN1, UQCRC1, NDUFS1, NDUFV2, COX6B2, COX7A1, SLC25A6
hsa00620:Pyruvate metabolism	4	5.50E-03	FH, PC, ACYP2, ACAT2
hsa04932:Non-alcoholic fatty liver disease	6	6.57E-03	NDUFA9, UQCRC1, NDUFS1, NDUFV2, COX6B2, COX7A1
hsa05208:Chemical carcinogenesis - reactive oxygen species	7	7.04E-03	NDUFA9, UQCRC1, NDUFS1, NDUFV2, COX6B2, COX7A1, SLC25A6
hsa05016:Huntington disease	8	8.66E-03	NDUFA9, POLR2A, UQCRC1, NDUFS1, NDUFV2, COX6B2, COX7A1, SLC25A6
hsa01200:Carbon metabolism	5	1.16E-02	FH, PC, GLYCTK, PCCB, ACAT2
hsa05020:Prion disease	7	1.79E-02	NDUFA9, UQCRC1, NDUFS1, NDUFV2, COX6B2, COX7A1, SLC25A6
hsa03320:PPAR signaling pathway	4	1.97E-02	CPT2, ACADL, EHHADH, HMGCS2
hsa00630:Glyoxylate and dicarboxylate metabolism	3	2.20E-02	GLYCTK, PCCB, ACAT2
hsa01240:Biosynthesis of cofactors	5	2.96E-02	GMPPB, ALAS2, SPR, FECH, MTHFD2
hsa00260:Glycine, serine and threonine metabolism	3	3.75E-02	ALAS2, GLYCTK, SARDH

GO, Gene Ontology. KEGG, Kyoto Encyclopedia of Genes and Genomes.
